# Decision of the Commissioner of Internal Revenue with Regard to the Income Tax to Be Paid by Physicians

**Published:** 1863-09

**Authors:** Joseph J. Lewis

**Affiliations:** Commissioner; Treasury Department, Office of Internal Revenue, Washington


TREASURY DEPARTMENT, OFFICE OF INTERNAL REVENUE,
WASHINGTON, June 11th, 1863.

DECISION OF THE COMMISSIONER OF INTERNAL REVENUE WITH REGARD TO THE INCOME TAX TO BE PAID BY PHYSICIANS.—Assessment should be made upon all collections during the year 1862, without regard to whether the services were rendered during that or previous years. If any profits made during that year and uncollected, remain uncollected when they might have been readily realized, and with a view merely to avoid the assessment of the tax, they are to be considered as collected, and assessed accordingly; for no evasion of the liabili y of the tax-payer of his duty under the law should be allowed to profit him. But merely contingent profits, uncollected, the sum not ascertained, remaining open for adjustment, are not liable to assessment.

2d. As to "expenses necessarily incurred in carrying on any trade, business or profession," physicians cannot be allowed the wear and tear of horses, carriages and harness, any more than they can of their own constitutions, or of their health, necessarily injured in the practice of their vocation; but any incidental expenses, such as the feeding of horses, hire of servants, and such like, are to be deducted from their income.

Very respectfully,

JOSEPH J. LEWIS, *Commissioner.*

